# The Effect of Changes in the Angular Position of Implants on Metal Artifact Reduction in Cone-Beam Computed Tomography Images: A Scoping Review

**DOI:** 10.1155/2023/5539719

**Published:** 2023-07-31

**Authors:** Maedeh Asnaashari, Maryam Sadeghipour, Zeinab Bahrani, Solmaz Valizadeh, Mahkameh Moshfeghi

**Affiliations:** ^1^School of Dentistry, Shahid Beheshti University of Medical Sciences, Tehran, Iran; ^2^Department of Community Oral Health, School of Dentistry, Shahid Beheshti University of Medical Sciences, Tehran, Iran; ^3^Department of Prosthodontics, School of Dentistry, Shahid Beheshti University of Medical Sciences, Tehran, Iran; ^4^Department of Oral and Maxillofacial Radiology, School of Dentistry, Shahid Beheshti University of Medical Sciences, Tehran, Iran

## Abstract

**Objective:**

Dental implant artifacts can compromise the quality of cone-beam computed tomography (CBCT) scans and challenge radiographic detection in surrounding regions. This literature review was conducted to examine the impact of implant angle modification on reducing metal artifacts in CBCT scans.

**Materials and Methods:**

A scoping review of literature was carried out in PubMed, Embase, Scopus, and Cochrane databases.

**Results:**

Different spatial planes, including alpha, beta, gamma, and phi, along with 0°, 5.2°, 9.8°, 14.5°, 15°, 30°, 45°, 60°, 75°, and 90° angles were studied. Changes in the angular position of implants may reduce metal artifacts and improve the quality of CBCT scans.

**Conclusions:**

Rotating implants within the alpha plane and angling them at 90° in the alpha plane enables reducing dental implant artifacts.

## 1. Introduction

Presence of artifacts, namely, spurious visual structures in cone-beam computed tomography (CBCT), can result in impaired image quality and detectability of radiographic details [1, 2]. Interference of absorbent materials (e.g., metals) with X-ray irradiation creates metal artifacts, deviating obtained scan details from true anatomy [1–3].

With the developing use of dental implants as the most common treatment form replacing lost teeth, CBCT has become the standard radiography for thorough pre- and post-operative evaluation of implants and surrounding anatomic structures. However, presence of metal artifacts degrades image quality and hinders assessment of adjacent structures [2]. Several studies have been conducted to surmount creation of artifacts or at least reduce their impact on image quality, focusing on the impact of different factors, including application of metal artifact reduction algorithm (MAR), using different implant materials, voltage, voxel size, tube current, the field of view (FOV), or spatial location of implants, etc. [4–32]. These factors have been observed to have minimal impacts on complete elimination of metal artifacts.

Given the scarce conclusive data regarding the plausible impact of changes in the angular position of implant while scanning on reducing metal artifacts in CBCT scans, this review study was carried out and an effort was made to answer the question whether changing the angular position of implant leads to metal artifact reduction or not.

As mentioned, metal artifacts decrease CBCT image quality and prevent evaluation of adjacent structures [2, 33, 34], and various factors may affect it [4–32]. One of those factors is changing the angle of implants [2, 33, 34]. Due to the importance of the issue, we decided to review the articles written on this topic.

## 2. Materials and Methods

### 2.1. Research Question

The scoping review question was, “How does the modification of the angular position of implants can reduce metal artifact in CBCT scans?”

### 2.2. Search Strategy

This scoping review is an evaluation of current literature, aiming to assess the evidence and detect existing gaps. A literature search was independently conducted by two individuals on PubMed, Embase, Scopus, and Cochrane databases from October 2022 until June 2023. The search strategy was (“dental implant” AND “cone beam computed tomography” AND “artifact”) with no additional filters. It should be noted that employed keywords were selected based on the MeSH terminology. The competency criteria were collected based on our research question.

### 2.3. Inclusion and Exclusion Criteria

Inclusion criteria entailed (1) availability of article full text and (2) investigation of the impact of modified angular position of implants on artifact reduction in CBCT scans. We excluded the studies based on the following exclusion criteria: (1) if they were failed to investigate the effect of angle change on reducing the artifacts caused by implants and (2) publication solely in non-English language.

### 2.4. Data Selection and Charting Process

**Figure 1:**
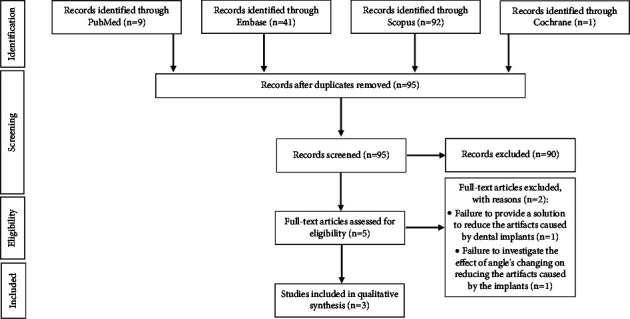
PRISMA-ScR flowchart. Flow chart of the studies' selection process.

The papers were gathered by performing an advanced search on the databases. To fulfil this goal, the keywords were separated with the Boolean operator “AND.” The search was completed using a manual search method. The gathered articles were screened three times. In the first screening, the databases were checked for copies of each paper. In the second screening, the papers were investigated based on their title and abstract. In this case, the papers that were not related to the topic were removed. In the third screening, the full texts of papers were reviewed and the screening process was carried out based on the exclusion criteria. After that, the chosen papers were synthesized according to their qualities. Then, the data of each chosen paper were extracted, including author's name, publication year, implant placement environment, number of implants, width of implants, length of implants, center-to-center distance between implants, anatomical region of the implants, material and brand of the implants, angles, exposure time, voltage, beam currents, study analysis, results, and conclusions. Thematic analysis was employed to analyze the collected data. This analysis is usually used to determine the themes of different topics, analysis, exegesis, description, and conclusion in a systematic review [35]. Article search process was shown in Figure 1. The characteristics and research results of the articles included in the study were given in Table 1. There were three articles to be studied in detail.

## 3. Results and Discussion

The purpose of this study was to review the impacts of changes in implant angular position on reducing metal artifacts in CBCT images. Reviewed articles were selected from the following databases: PubMed, Embase, Scopus, and Cochrane. The PRISMA-ScR flowchart showing the scoping review process is shown in Figure 1. Table 1 shows a summary of included articles, including author's name, year, implant placement environment, number of implants, width of implants, length of implants, center-to-center distance between implants, anatomical region of the implants, material and brand of the implants, angles, exposure time, voltage, beam currents, study analysis, results, and conclusions. Out of 143 articles, only three studies were included. Hence, more research is warranted to make clinical conclusions.

The effect of various factors on artifacts in CBCT images has been investigated, including metal artifact reduction algorithm (MAR), implant material, voltage, implant position, voxel size, tube current, and field of view (FOV) [5–21, 24, 25, 27–32, 36–39].

Many studies have observed MAR to be effective in reducing implant-induced artifacts [10, 11, 15, 17, 19–21, 27, 29, 32], whereas some reported MAR ineffective [5–7, 9, 13, 22, 24, 25, 28]. According to [26], implant material affects MAR efficacy. MAR is effective in reducing zirconia implant artifacts, while it fails to bar presence of titanium implant artifacts [26].

Evaluation of the effect of implant material on the number of artifacts has revealed that artifacts resulting from zirconium and titanium implant to hold the highest and least number of artifacts, respectively. There was a moderate chance of the creation of artifacts associated with titanium-zirconium implants [19, 29, 30, 32]. Kuusisto et al. reported that titanium implant artifacts were more common than glass fiber-reinforced composite implant artifacts [16].

Considering the voltage factor, all studies stated that an increase in kVp limited metal artifacts [11, 19, 29, 32].

Various studies have been conducted on implant position. Machado et al. postulated that artifacts appear more frequently in the mandible than maxilla, and artifacts appear more in the anterior areas than that of the posterior areas [12]. According to [31], artifacts most frequently occur around the incisors [31]. Moreover, Kocasarac et al. concluded that implants led to greater artifact expression in the exomass than inside the FOV [32].

Regarding the voxel size, previous studies have shown that smaller voxel size caused increased artifact production, while larger voxel size decreased the number of artifacts [11, 31]. The tube current (mA) factor had no impact on the number of artifacts according to Shokri et al. However, Mancini et al. showed that tube current increase was effective in reducing artifacts [18, 26]. Shokri et al.'s results were inconsistent with Mancini et al.'s in terms of FOV. According to Shokri et al., smaller FOV decreased artifacts. However, according to [31], smaller FOV increased artifacts in CBCT images [18, 31]. Several pertaining factors that can affect artifacts have also been examined. For example, according to Cortes et al., edited 3D-CBCT follow-up images of multiple implants minimized the number of artifacts [36].

Based on [30] the quantitative study, it was found that BAR (blooming artifact reduction) filter was the most effective among the four investigated filters (BAR and multi-CDT NR (noise reduction) filter in e-Vol DX Software and Filter 1x and Filter 2x in OnDemand software) in reducing artifacts and thus improving the quality of images [30]. Emami et al. revealed that the artifact removal (low-medium) option can reduce the number of artifacts caused by delicate structures like lamina dura, but it does not affect the number of artifacts caused by large anatomical structures and linear bone measurements [37]. According to [38], system automated motion artifact correction is useful in reducing artifacts [38]. Based on [19], resolution changes had no effect on the number of artifacts [19]. According to [20], adaptive image noise optimizer (AINO) optimization filter was effective in reducing artifacts [20]. In Cardarelli et al.'s study in 2021, they investigated a new protocol called “low-dose CBCT imaging protocol” which was useful in reducing artifacts [39]. Based on [27], increasing the number of basis images was effective in reducing artifacts [27].

In previous studies, the effect of “changes in the angular position of implants” on the reduction of metal artifacts was examined [2, 33, 34], so we decided to summarize this issue by conducting a review study. The hypothesis that different implant angles in patients with multiple implants can reduce metal artifacts can be justified by the beam hardening effect. Since by preventing the overlapping of these high radiation absorbing materials, beam hardening is reduced, improvement in image quality will occur [2, 33].

In Min and Kim's study in 2021, two dental implants were placed in a homogeneous dental impression material block. The block was scanned with a CBCT scanner at seven different angles (0°, 15°, 30°, 45°, 60°, 75°, and 90°) along three different planes. Rotation in the frontal plane was called alpha rotation. The second rotation in the sagittal plane was called beta rotation. The simultaneous rotation in the frontal and sagittal planes was called gamma rotation. Thirteen volumes of interest (VOI) were selected from each axial reconstruction. Gray values (GVs) of each VOIs were measured. Mean differences in GV between the control area and VOIs were calculated. These ΔGVs from different spatial angle were compared and analyzed by Welch's analysis of variance and linear regression. According to the research, increases in alpha angle increase ΔGVs of groups A and B but fail to significantly change group C. Furthermore, any notable changes in ΔGVs were observed for gamma rotation in all VOIs. The study concluded that an increase in alpha angle can reduce interimplant metal artifacts in CBCT images. Therefore, modification of the angle of the patient's head may lead to the reduction of metal artifacts [2].

The authors in [34] performed a study on alpha rotation, the rotation in the frontal plane. In this research, polyurethane synthetic bone blocks were first scanned without implants by CBCT and micro-CT (a gold standard for CBCT). Then, two dental implants were placed in the bone blocks and these blocks were scanned by CBCT at different alpha angles (0°, 15°, 30°, 45°, 60°, 75°, and 90°). Six microstructural parameters including bone volume per total volume, bone surface per total volume, trabecular spacing, trabecular thickness, fractal dimension, and connectivity were measured. Spearman correlation coefficients for each parameter from CBCT and micro-CT were calculated and compared using Steiger's *Z* test. Results manifested that for VOI1 and VOI2, as the alpha angle increases, the correlation coefficient increases; however, for VOI3, the correlation coefficient decreases with increasing alpha angle. Therefore, modifying the alpha angle can modify CBCT image quality [34].

In Luckow et al.'s study in 2011, two dental implants were placed in the mandible of a pig, and CBCT was performed based on varying the starting rotation angle of the mandible in the source-detector plane, beam current, accelerating voltage, and angles of the jaw with respect to the source-detector plane. In this research, rotations were performed in three different planes. These rotations were called alpha and beta rotations, respectively, which were performed in the frontal and sagittal planes. Simultaneous rotation in the frontal and sagittal planes was called phi rotation. In alpha and beta rotations, 0°, 5.2°, 9.8°, and 14.5° angles, and phi rotation, 0°, 30°, 45°, and 90° angles were examined. The different datasets were automatically registered with respect to micro-CT data to extract the common volume and the deviance to the predefined standard that characterizes the image quality. The research showed that image quality can be improved by increasing the *α* angle. Since the overlap of highly X-ray absorbing implants may reduce by tilting the jaw, tilting the lower jaw with dental implants can reduce artifacts in CBCT images [33].

In the three mentioned articles, the materials of the implants were the same and they were made of titanium. Also, quantification of artifacts was carried out in three articles and the method of measuring artifacts was quantitative [2, 33, 34]. As a result, comparing these articles together, the material of implants and method of measuring the artifacts were not considered interfering factors. The anatomical region of the implants in Luckow et al.'s study was in the mesial and distal of the canine [33], but in the other two articles, a bone block was used [2, 34], and due to the placement of the implants in the bone block, no equivalent can be considered for the exact location of the implant in the jaw. So, since this factor was not same in the articles, this factor can be considered an interfering factor in our study, and maybe the comparison of the results of the studies has a little problem due to this different index. We took this point into consideration, but unfortunately, there is no solution to solve it.

## 4. Conclusion

Based on the conclusion of three articles included in this study, among all the introduced plans (alpha, beta, gamma, and phi), alpha plane rotation was the most effective in artifact reduction and an increased alpha angle reduced the number of metal artifacts [2, 33, 34]. In summary, it seems that modification of implants' angle can reduce metal artifacts in CBCT images, improving the image quality consequent to head and jaw tilting which can reduce the overlap of implants and hard tissues with high x-ray absorption [2].

Given the small number of papers, as well as the laboratory nature of the studies, it is not possible to highly extend the conclusions to clinical works and more studies in this field are required; besides, more evidence must be collected and clinical research should be carried out.

## Figures and Tables

**Table 1:** Chart of included studies.

Author year reference	Implant placement environment	Number of implants	Width of implants (mm)	Length of implants (mm)	Center-to-center distance between implants (mm)	Anatomical region of the implants	Material and brand of the implants	Angles (degree)	Exposure time (seconds)	Voltage (kVp)	Beam currents (mA)	Study analysis	Result	Conclusion
Min and Kim 2021 [2]	A block made of polyvinyl siloxane impression material	Two	4	10	8.5	Due to the placement of the implants in the block, no equivalent can be considered for the exact location of the implant in the jaw	Titanium (point implant)	Alpha, beta, gamma 0 15 30 45 60 75 90	17	78	8	Quantitative analysis (metal artifacts were quantified using the mean and standard deviation of gray values)	With increasing alpha angle (rotation in the frontal plane), ΔGVs^*∗*^ of group A (VOIs^*∗∗*^ on the extension line of implants) and B (VOIs on the side of implants) increased, but no significant change was observed in group C (other peripheral VOIs). The ΔGVs of group A also increased with increasing beta angle (rotation in the sagittal plane). No significant change in ΔGVs for gamma rotation (simultaneous rotation in the frontal and sagittal planes) in all VOIs was observed	Alpha angle's increasing can reduce inter-implant metal artifacts in CBCT images and thus tilting patient's head is a way to reduce metal artifacts

Min and Kim 2020 [34]	Polyurethane synthetic bone	Two	4	10	8.5	Due to the placement of the implants in the bone block, no equivalent can be considered for the exact location of the implant in the jaw	Titanium (point implant)	Alpha 0 15 30 45 60 75 90	17	78	8	Quantitative analysis (correlation coefficients of microstructural parameters in micro-CT and CBCT were used to quantify the effect of metal artifacts)	For VOI_1_ (the VOI between the implants) and VOI_2_ (the VOI on the extension line of the two implants), the correlation coefficient increased with increasing alpha angle (rotation in the plane that including the longitudinal axes of the two implants) from zero to 90°. Unlike other VOIs, for VOI_3_ (the VOI on the side-of the extension line), the correlation coefficients gradually decreased with increasing alpha angle	Alpha angle's changes can affect the quality of CBCT image

Luckow, et al. 2011 [33]	Lower jaw of a five-month-old pig	Two	4.1	10		Mesial and distal of the canine	Titanium (straumann AG)	Alpha, beta 0 5.2 9.8 14.5 Phi 0 30 45 90	17	70 72 74 76 78 80 (70 to 80)	2 7 10 (1 to 10)	Quantitative analysis (the deviance of the detected local absorption values (gray levels) between the CBCT-data and the (SR) CT-data were used to quantify the image quality)	Tilting the pig's mandible in the frontal plane called *α* angle. Increasing the *α* angle can improves the image quality	Moderate tilting of the porcine mandible with dental implants can reduce the artifacts in the CBCT images and improve the image quality because tilting the jaw can reduce the overlap of highly X-ray absorbing implants and hard tissues

^*∗*^ΔGVs: mean gray values, ^*∗∗*^VOIs: volumes of interest.

## Data Availability

The study's findings were supported by previously reported data in the studies and cited datasets.
